# Impact of DNA extraction, PCR amplification, sequencing, and bioinformatic analysis on food-associated mock communities using PacBio long-read amplicon sequencing

**DOI:** 10.1186/s12866-024-03677-8

**Published:** 2024-12-06

**Authors:** Mareike Baer, Lisa Höppe, Waldemar Seel, André Lipski

**Affiliations:** 1 Institute of Nutritional and Food Sciences, Food Microbiology and Hygiene, University of Bonn, Friedrich-Hirzebruch-Allee 7, 53115 Bonn, Germany; 2 Institute of Nutritional and Food Sciences, Nutrition and Microbiota, University of Bonn, Katzenburgweg 7, 53115 Bonn, Germany

**Keywords:** PacBio sequencing, Full-length 16S rRNA gene, Long-read sequencing, Amplicon sequencing, Mock community, Food microbiota

## Abstract

**Background:**

Long-read 16S rRNA gene amplicon sequencing has a high potential for characterizing food-associated microbiomes. The advantage results from sequencing the full-length (1,500 bp) gene, enabling taxonomic resolution at species level. Here we present a benchmarking study using mock communities representative of milking machine biofilms and raw meat, revealing challenges relevant to food-associated habitats. These were varying species abundances, reliable intra-genus differentiation of species, and detection of novel species with < 98.7% sequence identity to type strains. By using mock communities at different levels of preparation − as mixed whole cells, mixed extracted DNA, and mixed PCR products − we systematically investigated the influence of DNA extraction using two different kits, PCR amplification of 16S rRNA genes, sequencing, and bioinformatics analysis including reference database and gene copy number normalization on bacterial composition and alpha diversity.

**Results:**

We demonstrated that PacBio ccs-reads allowed for correct taxonomic assignment of all species present within the mock communities using a custom Refseq database. However, choice of percent identity values for taxonomic assignment had a strong influence on identification and processing of reads from novel species. PCR amplification of 16S rRNA genes produced the strongest bias on the observed community composition, while sequencing alone reproduced the preset composition well. The PCR bias can in part be attributed to differences in mol% G + C content of 16S rRNA genes resulting in preferred amplification of low mol% G + C-containing taxa.

**Conclusions:**

This study underlines the importance of benchmarking studies with mock communities representing the habitat of interest to evaluate the methodology prior to analyzing real samples of unknown composition. It demonstrates the advantage of long-read sequencing over short-read sequencing, as species level identification enables in-depth characterization of the habitat. One benefit is improved risk assessment by enabling differentiation between pathogenic and apathogenic species of the same genus.

**Supplementary Information:**

The online version contains supplementary material available at 10.1186/s12866-024-03677-8.

## Background

Many food products contain a highly diverse microbiota considerably affecting the characteristics of the product. The microbial metabolic activity affects shelf-life, but may also increase product quality by synthesis of stabilizing and flavoring compounds. Detailed knowledge about food microbial composition is pivotal for shelf-life prediction, risk assessment, but also quality control and improvement of the food product. Besides traditional cultivation techniques, next-generation sequencing (NGS) methods are increasingly used to characterize food microbiomes [1]. They involve total DNA extraction, optional PCR amplification of marker genes, e.g. bacterial 16S rRNA genes, followed by high-throughput sequencing (HTS). The advantage of these methods compared to cultivation are economy of time and independence of incubation conditions (e.g. nutrients, temperature, atmosphere), combined with the possibility to detect viable but nonculturable (VBNC) cells [2, 3]. The drawback of detecting DNA of dead cells can be overcome by using viable PCR (vPCR) [4–6]. This method applies the DNA intercalating dye propidium monoazide (PMA), which only penetrates damaged cells and forms covalent bounds with DNA upon light induction. PMA-modified DNA is excluded from subsequent PCR-amplification [7].

Amplicon sequencing is a method frequently applied in food microbiology to characterize microbial communities of various foodstuff ranging from dairy products and surfaces [8, 9] to meat [2, 10], vegetables [11, 12], and fermented foods and beverages [13, 14]. Many studies aim on tracing contamination sources within food production facilities [8, 15], or tracking the temporal development of the microbiota during processing or ripening [13, 16]. Other purposes are risk assessment, i.e. detection of pathogens, and characterization of microbes with valuable functions [1, 17]. The vast majority of studies rely on short-read sequencing of different hypervariable regions of the 16S rRNA gene (≈ 450 bp) sequenced by Roche 454 (discontinued in 2016), Illumina MiSeq, or Ion PGM technologies [18]. Sequencing of less than one third of the 16S rRNA gene’s length often only allows for taxonomic classification at the family or genus level [19]. However, many genera comprise both pathogenic and non-pathogenic species, making risk assessment from these data difficult [20, 21]. Moreover, choice of primer pairs might impair detection of certain taxa, or produce false positives due to close relatives [11, 18]. This becomes clear, as there is no consensus on the most suitable region to sequence, although the V3-V4 region seems to perform best [22–24]. Third-generation sequencing platforms such as Oxford Nanopore (ONT) and Pacific Biosciences (PacBio) produce remarkably longer reads, allowing for full-length (1,500 bp) 16S rRNA gene sequencing. This eliminates bias resulting from sequencing of different hypervariable regions. Moreover, taxonomic resolution is improved, allowing for identification at species level. Since their first implementation, various efforts have been made to overcome their property of producing more sequencing errors than short-read sequencers. One strategy is the circular consensus sequencing (ccs) workflow in SMRT Link for the PacBio Sequel platforms generating HiFi reads with 99.9% accuracy [19, 22].

Benchmarking studies using mock communities of known composition are recommended for NGS approaches to evaluate technical bias, and have been performed by many studies. These studies focus on aspects such as choice of DNA extraction method, primer combinations and library preparation, or comparison of sequencing platforms and/or bioinformatic pipelines including reference databases [22, 24, 25]. Mock communities are constructed from representatives of the habitat of interest, e.g. the human gut [18, 26], agricultural environments [25], and food [20, 27]. Alternatively, commercial mock communities are available and have been applied as reference (e.g. from the American Type Culture Collection, ATCC, or Zymo Research) [24, 25]. Some studies used DNA mixtures instead of whole-cell mock communities [16].

PacBio 16S amplicon sequencing technology is only beginning to be applied for assessing food microbial composition [15, 17, 28, 29]. Thus, the first aim of the present study was to assess the suitability of this technology for sequencing mock communities representative of two food-associated habitats, i.e. milking machine biofilms and raw meat (beef and chicken). Mock communities were generated to represent realistic scenarios present in food. These are (a) dominance or minority of single species; (b) presence of taxonomically closely related species of the same genus; (c) presence of yet undescribed species displaying less than 98.7% identity to known type strains represented in databases. The second aim of the study was to systematically evaluate the effects of DNA extraction using two different kits, PCR amplification, and sequencing on bacterial community composition by comparing observed to expected species abundances. Besides extracting DNA from whole-cell mock communities, DNA extracts from single cultures were mixed to assess the impact of PCR amplification on bacterial community composition. Finally, 16S rRNA genes were amplified from single cultures, and PCR products were mixed before sequencing to assess the bias produced by the sequencing procedure itself. Diverse parameters were used in the bioinformatics pipeline Qiime2 [30] to present the impact of analysis procedures on the observed bacterial composition. To our knowledge, this is the first benchmarking study systematically assessing the impact of DNA extraction, PCR amplification of full-length 16S rRNA genes, sequencing, and bioinformatic data processing on bacterial composition and alpha diversity of two different food-associated mock communities using long-read PacBio sequencing.

## Methods

### Mock community composition

Mock communities representative of two different food-associated habitats were defined using bacterial isolates originating from (a) milking machine biofilms and (b) raw meat (beef and chicken). The milking machine biofilm community composition has been characterized in a previous study [5], while representative meat isolates were chosen according to cultivation experiments conducted in our lab (unpublished). All isolates were identified by sequencing the almost complete 16S rRNA gene. Table 1 lists the 13 biofilm and 14 meat isolates used for mock community construction together with relevant properties of each species (i.e. sequence identity to reference strains, number of 16S rRNA gene copies per cell, genome size, and mol% G + C content of the 16S rRNA genes).

**Table 1:** Overview of mock community bacterial composition and genomic attributes of species. Genomic attributes of reference genomes were obtained from the NCBI Datasets Genome website (https://www.ncbi.nlm.nih.gov/datasets/genome/) [31]. 16S rRNA gene copy number derived from the rrnDB [32] version 5.6 automatically matched by the q2-gcn-norm plugin [33]. If no match was found for the taxon assigned on species level, the script matched higher ranks and assigned the mean copy number of all respective database entries. *Bac*, *Bacillota*; *Act*, *Actinomycetota*; *Pseu*, *Pseudomonadota*; *Bact*, *Bacteroidota*. Div, diverging bases; l, alignment length

Origin	Phylum / Species		Strain	% ID with Refseq (div/l) ^1)^	16 S rRNA gene copies	Genome size (Mb)	16 S rRNA gene G + C content (mol%)
**Biofilm isolates**	*Bac*	*Bacillus pumilus*	M11	99.9 (1/1471)	7.9 ^2)^	3.6	55.2
		*Enterococcus faecalis*	M249	99.8 (3/1483)	4	2.9	54.0
		*Lacticaseibacillus paracasei*	M57	99.9 (2/1492)	5	3.0	53.1
		*Staphylococcus cohnii*	M34	99.9 (0/1475)	6	2.7	51.3
	*Act*	*Dermacoccus nishinomiyaensis*	M293a	99.7 (3/1436)	3	3.1	57.4
		*Gordonia paraffinivorans*	M69	99.8 (3/1443)	3.3 ^2)^	4.5	57.9
		*Kocuria salsicia*	M239	99.8 (3/1426)	3	3.1	57.3
		*Microbacterium oxydans*	M29	99.1 (8/1438)	2	3.9	56.0
	*Pseu*	*Acinetobacter guillouiae*	M9	99.5 (9/1461)	7	4.6	52.7
		*Pseudomonas koreensis*	M92	99.8 (3/1455)	5.5 ^2)^	6.1	53.7
		*Escherichia coli*	M159	99.7 (1/1464)	7	4.6	54.9
		*Stenotrophomonas lactitubi*	M15	100 (0/1469)	3.4 ^2)^	4.9	54.9
	*Bact*	*Chryseobacterium shigense*	M169	99.3 (5/1440)	3.7 ^2)^	4.9	50.3
			**Mean (standard deviation)**		4.8 (1.8)	4.0 (1.0)	54.5 (2.3)
**Meat isolates**	*Bac*	*Brochothrix thermosphacta*	RK8b	99.7 (5/1421)	9	2.6	53.1
		*Staphylococcus condimenti*	H13a	99.8 (1/1474)	5.7 ^2)^	2.7	52.6
	*Act*	*Rothia nasimurium*	H14a	99.7 (4/1451)	3	2.5	56.2
	*Pseu*	*Serratia quinivorans*	H25	99.5 (7/1465)	7	5.4	54.5
		*Acinetobacter guillouiae*	R7a	99.5 (3/1463)	7	4.6	53.0
		*Proteus mirabilis*	H1	99.7 (3/1465)	6.8 ^2)^	4.1	53.0
		*Pseudomonas weihenstephanensis*	RK8a	99.9 (2/1459)	5.6 ^2)^	4.8	53.1
		*Pseudomonas petroselini*	HK3a	99.6 (5/1459)	5.6 ^2)^	6.9	53.6
		*Pseudomonas helleri*	HK4	99.5 (8/1459)	5.6 ^2)^	5.7	53.0
		*Pseudomonas gessardii*	H4a	99.9 (2/1459)	5.6 ^2)^	6.5	53.5
		*Citrobacter gillenii*	R22	99.9 (0/1464)	7.8 ^2)^	5.2	54.6
		*Acinetobacter spp. (pullicarnis)*	R25a	98.2 (21/1462)	6.7 ^2)^	4.1	52.7
		*Pantoea agglomerans*	H6a	99.0 (14/1461)	7	4.7	55.5
	*Bact*	*Chryseobacterium shigense*	H9a	99.2 (6/1440)	3.7 ^2)^	4.9	50.2
			**Mean (standard deviation)**		6.2 (1.5)	4.6 (1.4)	53.5 (1.4)

^1)^ BLAST [34] alignment of the isolate sequence and the best-hit sequence derived from the custom Refseq database. Diverging bases compared to alignment length are given in parentheses

^2)^ Genus level mean 16S rRNA gene copy number derived from the rrnDB [32]

Mock communities were composed of equal volumes of pure cultures grown in TSB at 30 °C overnight and adjusted to an OD_600_ of 0.1, corresponding to approximately 8 log_10_ cfu/ml. For biofilm isolates two independent mock communities were analyzed as biological replicates at two different time points, further referred to as mock community (MC) A and B. The meat mock community is referred to as MC C. Additionally, mock communities with high over- or underrepresentation of a particular bacterial strain were prepared to determine the effect on the community analysis. Mock communities D and E are identical with MC B except for the population of the isolate *Acinetobacter guillouiae* M9, which was diluted by the factor of 10^− 4^ in MC D, while MC E contained an undiluted *A. guillouiae* M9 population mixed with 10^− 4^ dilutions of all other biofilm isolates. To determine the actual cell number of each isolate within the mock communities, cultivation counts of the OD_600_ adjusted cultures were determined on tryptic soy agar (TSA) or MRS agar under anaerobic incubation for lactic acid bacteria (LAB). A spiral diluter (Interscience, Roubaix, France) was used with the 10^− 4^ dilutions as input material. However, colony counts of *Lacticaseibacillus paracasei* M57 and *Dermacoccus nishinomiyaensis* M293a in MC A and B, *Chryseobacterium shigense* M169 in MC B, and *Rothia nasimurium* H14a in MC C were below the detection limit of 6.6 log_10_ cfu/ml. For these samples, the cell count of 6.6 log_10_ cfu/ml was used for calculating preset relative abundance. Moreover, colony counts of *Proteus mirabilis* H1 were not determined owing to the swarming behavior of this strain. Preset relative abundances of this strain were calculated based on 8 log_10_ cfu/ml.

### DNA extraction and PCR amplification

Two different commercial kits were used for DNA extraction from mixed cultures. The Monarch^®^ HMW DNA Extraction Kit for Tissue by New England Biolabs (NEB, Ipswich, USA) will further be referred to as kit 1. The second kit was the Qiagen DNeasy Blood & Tissue Kit (Qiagen, Hilden, Germany), further referred to as kit 2. To standardize cell disruption, we used our in-house lysis buffer (20 mM Tris HCl (pH 8.2), 2 mM EDTA, 1.2% Triton-X-100) containing 25 mg/ml of freshly added lysozyme (PanReac AppliChem, Darmstadt, DE). After the lysis step at 37°C for 30 min, DNA was extracted according to the manufacturers’ protocols, which were the low-input protocol for Gram-positive bacteria (kit 1), and the protocol for Gram-positive bacteria (kit 2). All reactions involved RNAse A treatment. DNA of pure cultures was extracted using kit 2. Bacteria-specific primers with 16 bp barcodes were used to amplify the full-length 16S ribosomal RNA gene (V1-V9) using KAPA HiFi HotStart ReadyMix (Roche, Basel, CH). Primers included dual unique barcodes and a degenerate site-specific sequence: 27F (5’-barcode-AGRGTTYGATYMTGGCTCAG-3’) and 1492R (5′-barcode- RGYTACCTTGTTACGACTT-3′). The following cycling conditions were used for PCR: 95 °C for 3 min, followed by 23 cycles of 95 °C for 30 s, 57 °C for 30 s and 72 °C for 60 s. Following length verification on 1% agarose gels, PCR products were purified using AMPure PB beads (Pacific Biosciences, CA, USA) according to the recommended protocol.

DNA extraction, PCR amplification, and subsequent sequencing represents the standard protocol for characterizing food samples. Consequently, ten samples (M1-M10) were produced from whole cell mock communities MC A-E using two different DNA extraction kits and included all three factors extraction/PCR/sequencing (E/P/S) at the same time. To evaluate the impact of PCR/sequencing (P/S) and sequencing (S) alone, we constructed additional samples eliminating one factor at a time. Samples M11 to M14 (MC F-I) were assembled from DNA extracted from pure cultures, and represent the factors PCR/sequencing. DNA concentrations were measured using the Take3 Microvolume Plate in a microplate reader (Agilent BioTek, Schwerte, Germany), and adjusted to 2 ng/µl. Equal volumes were mixed representing biofilm (MC F) and meat (MC G) communities, respectively. Additionally, DNA extracts of pure culture biofilm isolates were diluted by the factor of 10^− 4^. MC H contained diluted DNA from *A. guillouae* M9 (10^− 4^, 20 pg/µl) mixed with DNA (2 ng/µl) of all other biofilm isolates, while MC I contained DNA of *A. guillouiae* M9 (2 ng/µl) mixed with the 10^− 4^ dilutions (20 pg/µl) of all other isolates, mimicking under- and overrepresentation of this species, respectively. Mixed DNA extracts were PCR amplified using amplicon primers as described above. Moreover, DNA extracts of single cultures were amplified separately using the same adapter primers (for biofilm and meat isolates, respectively), and PCR products were purified as described earlier. The concentrations of these purified PCR products were equalized and equal volumes were mixed prior to sequencing. These combined samples, labelled M15 (MC J) for biofilm and M16 (MC K) for meat, were prepared to test the effect of sequencing alone. A detailed description of all samples is presented in Table 2.

### Sequencing and bioinformatics processing

Amplicons were quantified using Qubit dsDNA HS Assay Kit from Thermo Fisher Scientific (Waltham, MA, USA) and fragment size was checked on a D1000 ScreenTape (Agilent Technologies, Santa Clara, CA, USA). The HiFi SMRTbell library was prepared using the SMRTbell Express Template Prep Kit 2.0 (Pacific Biosciences, CA, USA) together with Sequencing Primer v4 according to the manufacturer’s protocol. The library was sequenced on a PacBio Sequel I sequencer using Pacific Biosciences Sequel Binding kit v3 sequencing chemistry with a movie collection time of 10 h and a pre-extension time of 1.3 h on SMRT Cell 1 M. The final loading concentration was 6 pM. Demultiplexing of PacBio sequencing data and subsequent generation of HiFi reads was performed using SMRT Link software version 10.1. Demultiplexing was based on a unique barcode sequence combination. HiFi reads were generated to produce highly accurate (HiFi) consensus sequences. HiFi reads are generated using Circular Consensus Sequencing (CCS) technology, which iteratively sequences the same DNA molecule multiple times to correct errors and produce highly accurate consensus reads.

All bioinformatics analyses were conducted using the Qiime2 Amplicon distribution 2023.9 [30]. Demultiplexed ccs-reads were imported as fastq-files. Samples were denoised using the DADA2 command “denoise ccs” with default parameters. Denoising included quality filtering, primer and length (1200–1600 bp) trimming and chimera checking. Features were filtered for occurrence in ≥ 2 samples in a frequency of ≥ 100. Filtered features were removed from the sequences file prior to further processing. Due to higher amplicon sequence variant (ASV) counts than expected by mock community composition, *de novo* operational taxonomic unit (OTU)-clustering was conducted to reduce the amount of features. An identity of 98.7% was used for OTU-clustering, corresponding to the cutoff for species delineation [35]. The value was adjusted to 99.5% due to high identities of sequences within the meat mock community samples. A custom 16S rRNA gene sequence database was created by downloading 24,925 Refseq [36] 16S sequences (≥ 1200 bp) from the NCBI website, importing them into Qiime2, and using the feature-classifier “makeblast db” command. Taxonomic assignment of mock community samples was subsequently conducted using the “feature-classifier classify-consensus-blast” [37] command with the custom database. The maxaccepts value was set to one due to difficulties in species assignment when accepting more than one hit. The identity value for taxonomic assignment was set to 98.7%, as mentioned earlier. As one isolate within the meat mock community displayed ≤ 98.2% sequence identity to the type strains contained in the database (Table 1), a value of 97% was additionally used for the respective samples. In addition to the *de novo* clustered data, taxonomic assignment was also conducted for the denoised only data. After taxonomic classification, abundance of each feature was normalized on the basis of 16S rRNA gene copies using the plugin “gcn-norm copy-num-normalize” [33]. The plugin matches the taxonomic assignment with the copy number of 16S rRNA genes derived from the rrnDB database [32] version 5.6. If no match is found for the taxon assigned at species level, the script matches higher taxonomic ranks (e.g. genus) and assigns the mean copy number of all database entries for the respective genus. Absolute taxa abundances are then divided by the amount of 16S rRNA gene copies. The respective samples will further be referred to as normalized (norm) samples.

**Fig. 1:**
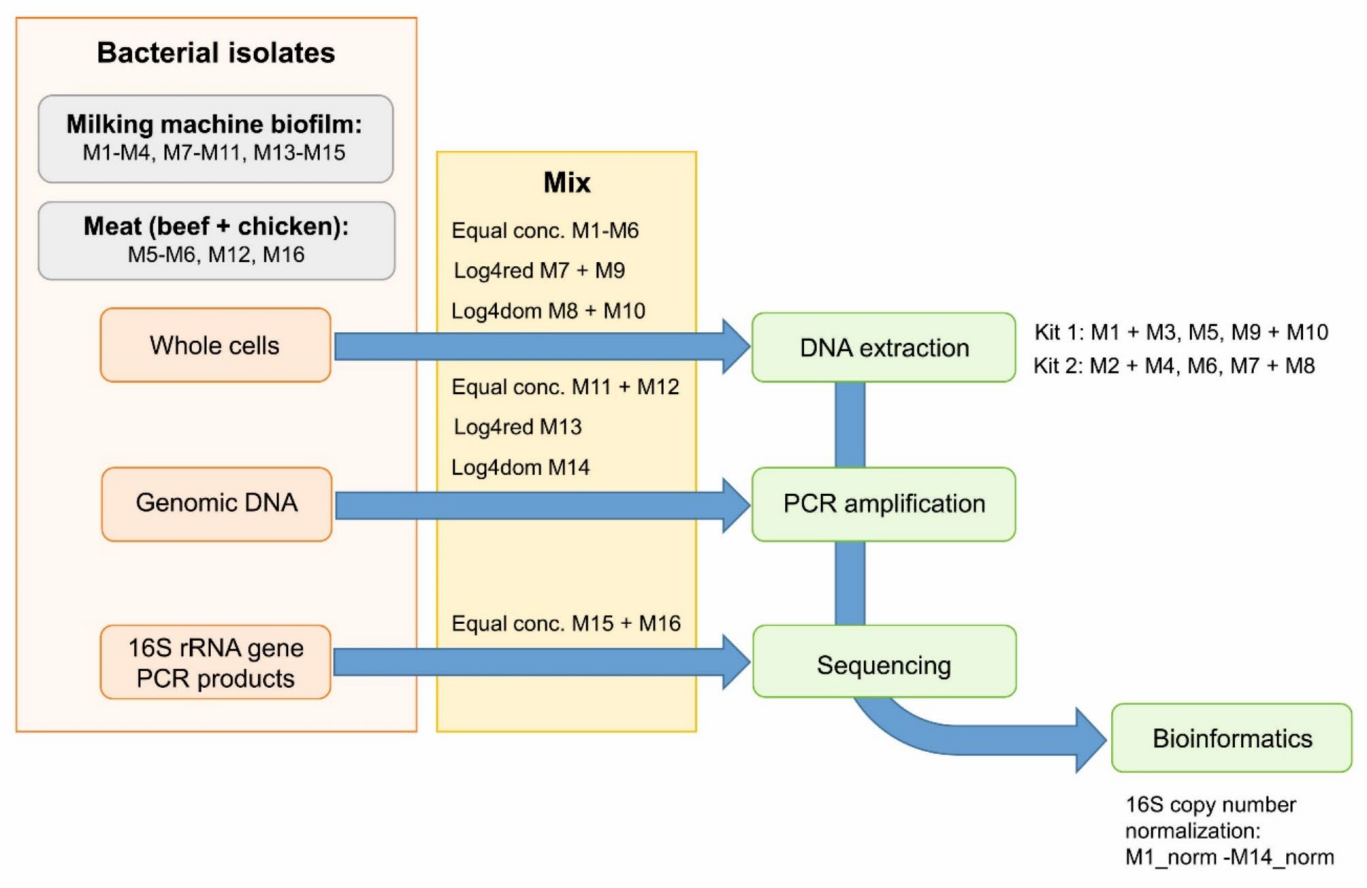
Visual representation of mock community processing. Conc, concentration; log4red, dilution of *Acinetobacter guillouiae* M9 by the factor of 10^− 4^; log4dom, dominance of *A. guillouiae* M9 by the factor of 10^4^; norm, correction (normalization) for 16 S copy numbers

Relative taxa abundances were visualized using the “taxa barplot” command, while absolute frequencies were visualized using heatmaps. To determine the effect of PCR amplification alone, abundances resulting from DNA mixtures of isolates (samples M11-M14) were additionally normalized by genome size per species. Sizes of reference genomes of the respective species were obtained from the NCBI Datasets Genome website (https://www.ncbi.nlm.nih.gov/datasets/genome). Preset abundances were calculated from cultivation counts. Log2 fold changes of preset versus observed abundances per species were calculated to determine the effect of DNA extraction using different kits, PCR amplification, and sequencing. A phylogenetic tree was generated from the sequences of all samples using “phylogeny align-to-tree-mafft-fasttree”. The resulting rooted tree was further used as input for describing alpha diversity by using “diversity core-metrics-phylogenetic” with an input sampling depth of 500. The sampling depth of 500 was chosen based on the frequency table visualization, aiming to retain all samples in the analysis. Alpha diversity metrics considered for the study were Pielou’s Evenness (J) assessing the uniform distribution of species within a sample, as well as Shannon Entropy (H) and Faith PD as measures of diversity. In contrast to Shannon Entropy, Faith PD takes into account phylogenetic relatedness of detected taxa. Overall observed features were also regarded. Rarefaction analysis was conducted using different rarefication depths (150–5000). The effects of 16S copy number normalization, DNA extraction, PCR amplification, sequencing, dilution of *A. guillouiae*, and origin of isolates on alpha diversity measures was depicted by boxplots. Significance of differences between groups was assessed by Kruskal Wallis pairwise tests, p-values were FDR corrected by the Benjamini & Hochberg method. Visualizations were generated using Microsoft Excel (v2016) or R (4.3.2) [38] using the ggplot2 package [39]. Figure 1 visualizes the procedures conducted with different mock communities and lists corresponding samples.

## Results

Table 2 summarizes sample-IDs and properties of the respective samples (origin of isolates, kit used for DNA extraction, input material, dominance/dilution of *A. guillouiae* M9, 16S rRNA gene copy number normalization). Samples M1-M11 assessed the impact of DNA extraction, PCR amplification and sequencing (E/P/S), M12-M14 assessed PCR amplification and sequencing (P/S), and M15-M16 assessed sequencing (S) alone. Absolute read frequency after quality and length filtering, denoising and chimera-removal, as well as alpha diversity measures for a sampling depth of 500 are additionally included in Table 2. Detailed denoising statistics can be taken from Supplementary Table 1. Unless otherwise stated, normalized samples are further referred to as 16S rRNA gene copy number corrected data. Between 46.6% (M12) and 76.7% (M1) of input reads passed the denoising process. Read frequency prior to normalization for 16S rRNA gene copies ranged from 3,481 (M5) to 55,397 (M4), which was reduced to 8.5% (M12) to 16.8% (M1, M7) of input reads by 16S gene copy number normalization. M2 had the lowest overall frequency of 509 after copy number normalization, which determined sampling depth of 500 for alpha diversity analysis. Low read frequencies did not affect observed diversity of the respective samples: Shannon Entropy (H) and Pielou’s Evenness (J) values were higher for M5 than for M6. While M5 was the sample with the lowest frequency after normalization, M6 was extracted from the same mock community using a different kit, and had the highest read frequency of meat mock community samples.

**Table 2:** Sample characteristics and alpha diversity measures. norm., normalized; n.a., not applicable; conc., concentration; red, reduction of *Acinetobacter guillouiae* by the factor of 10^−4^; dom, dominance of *Acinetobacter guillouiae*

Sample-ID	Origin	DNA extraction kit	Factor	Input material	Mock community (MC)	log_10_ fraction of *A. guillouiae*	16 S copy number normalization	Read frequency ^1)^	Pielou’s Evenness (J)	Shannon Entropy (H)	Faith PD	Observed Features
									Sampling depth: 500			
M1	Biofilm	1	DNA extraction/PCR/Sequencing (E/P/S)	Mixed culture (equal OD_600_)	A	1	raw	13,972	0.89	3.28	1.36	13
M1_norm							norm.	3055	0.89	3.29	1.36	13
M2		2					raw	5281	0.86	3.18	1.36	13
M2_norm							norm.	1166	0.85	3.14	1.36	13
M3		1			B		raw	19,415	0.82	2.84	1.26	11
M3_norm							norm.	4065	0.79	2.82	1.31	12
M4		2					raw	55,397	0.83	3.09	1.36	13
M4_norm							norm.	12,346	0.82	3.03	1.36	13
M5	Meat	1			C		raw	3481	0.85	3.31	1.19	15
M5_norm							norm.	509	0.90	3.50	1.19	15
M6		2					raw	44,686	0.81	3.17	1.19	15
M6_norm							norm.	6452	0.87	3.38	1.19	15
M7	Biofilm	2		Mixed culture (log4red)	D	− 4	raw	46,564	0.85	2.93	1.26	11
M7_norm							norm.	10,680	0.83	2.89	1.22	11
M8				Mixed culture (log4dom)	E	4	raw	53,685	0.02	0.02	0.39	2
M8_norm							norm.	7686	0.03	0.04	0.55	3
M9		1		Mixed culture (log4red)	D	− 4	raw	27,648	0.80	2.66	1.17	10
M9_norm							norm.	6305	0.74	2.65	1.27	12
M10				Mixed culture (log4dom)	E	4	raw	45,294	0.02	0.02	0.39	2
M10_norm							norm.	6477	0.02	0.02	0.37	2
M11	Biofilm	n. a.	PCR/Sequencing (P/S)	Pure culture DNA (equal conc.)	F	1	raw	19,100	0.82	2.94	1.31	12
M11_norm							norm.	4646	0.84	3.02	1.31	12
M12	Meat				G		raw	17,146	0.78	2.90	1.17	13
M12_norm							norm.	3125	0.71	2.64	1.17	13
M13	Biofilm			Pure culture DNA (log4red)	H	− 4	raw	21,918	0.83	2.97	1.27	12
M13_norm							norm.	5612	0.86	2.98	1.22	11
M14				Pure culture DNA (log4dom)	I	4	raw	55,135	0	0	0.19	1
M14_norm							norm.	7880	0.02	0.02	0.41	2
M15			Sequencing (S)	Pure cultures PCR products (equal conc.)	J	1	n. a.	13,400	0.98	3.64	1.36	13
M16	Meat				K	1		17,316	0.96	3.73	1.19	15

^1)^ Frequency after quality and length filtering, denoising and chimera-removal

**Fig. 2:**
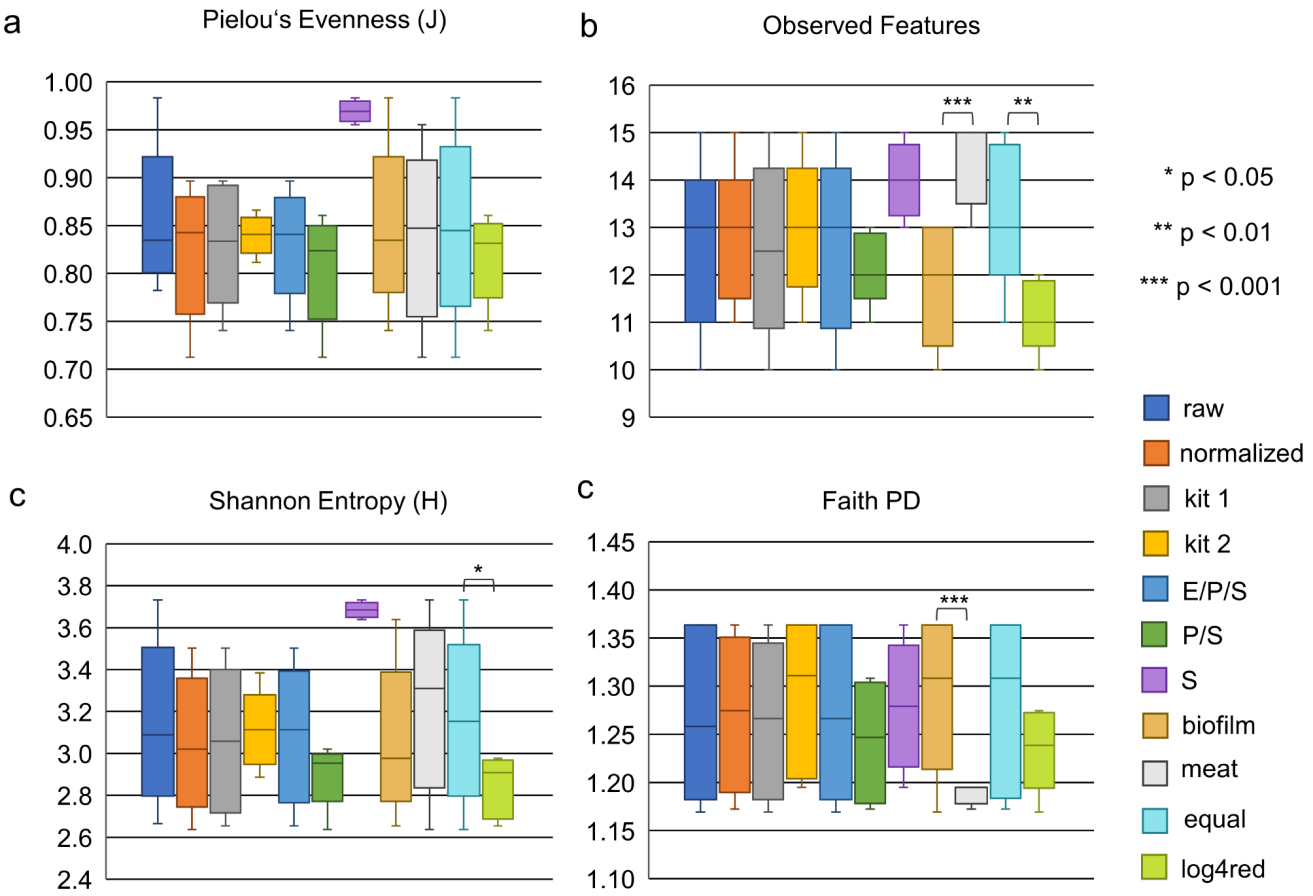
Influencing factors on alpha diversity measures of biofilm and meat mock communities. **a**, Pielou’s Evenness; **b**, Observed Features; **c**, Shannon Entropy; **d**, Faith PD. Significance of differences between groups was assessed by Kruskal Wallis pairwise tests, p-values were FDR-corrected by the Benjamini & Hochberg method. OTU-clustering was conducted at 98.7% identity for biofilm samples, while a cutoff of 99.5% was used for meat samples. Raw, not corrected for 16S rRNA gene copy number; equal, uniform concentration of all mock community members; E/P/S, P/S combined factors DNA extraction (E), PCR amplification (P), and sequencing (S); log4red (log 4 reduction), dilution of isolate *Acinetobacter guillouiae* M9 by the factor of 10^− 4^

Figure 2 demonstrates that samples which were only affected by the factor sequencing displayed higher values for Evenness (J) and Shannon Entropy (H) compared to the combined factors DNA extraction/PCR/sequencing (E/P/S) and PCR/sequencing (P/S) (Fig. 2A, C), although the differences were insignificant (*p* = 0.06 and 0.07 for Shannon Entropy, *p* = 0.08 and 0.1 for Evenness). Pielou’s Evenness (J) assesses the uniform distribution of species within a sample (1.0 = completely uniform, 0 = dominance of a single species). Shannon Entropy (H) is a measure of diversity, where higher values represent higher diversity. The highest possible Shannon Entropy values were 3.70 for biofilm and 3.81 for meat mock communities at equal relative abundance of all isolates. The indication of 15 observed features in the meat mock community samples (Table 2; Fig. 2) resulted from the detection of two separate features of different length, both representing the species *Pantoea agglomerans*. A significant difference (*p* < 0.01) was detected between observed features in equally concentrated samples vs. log 4 reduction (log4red) samples containing *A. guillouiae* in 1:10^− 4^ dilution (Fig. 2B). As meat mock communities contained four different species of the genus *Pseudomonas*, and Faith PD takes into account phylogenetic relatedness, a highly significant difference (*p* < 0.001) was detected between biofilm and meat mock communities (Fig. 2D). Low Faith PD values point to a higher phylogenetic relatedness of community members. No significant differences of alpha diversity measures were observed between raw and 16S copy number normalized samples, and between different DNA extraction kits (Fig. 2AD). However, since alpha diversity evaluates within sample diversity, equal values do not allow conclusions on equal species composition of different samples. This becomes clear, as Evenness (Fig. 2A) and Shannon Entropy (Fig. 2C) did not differ significantly between biofilm and meat mock communities, although their community composition at species level is completely different (Table 1).

**Fig. 3:**
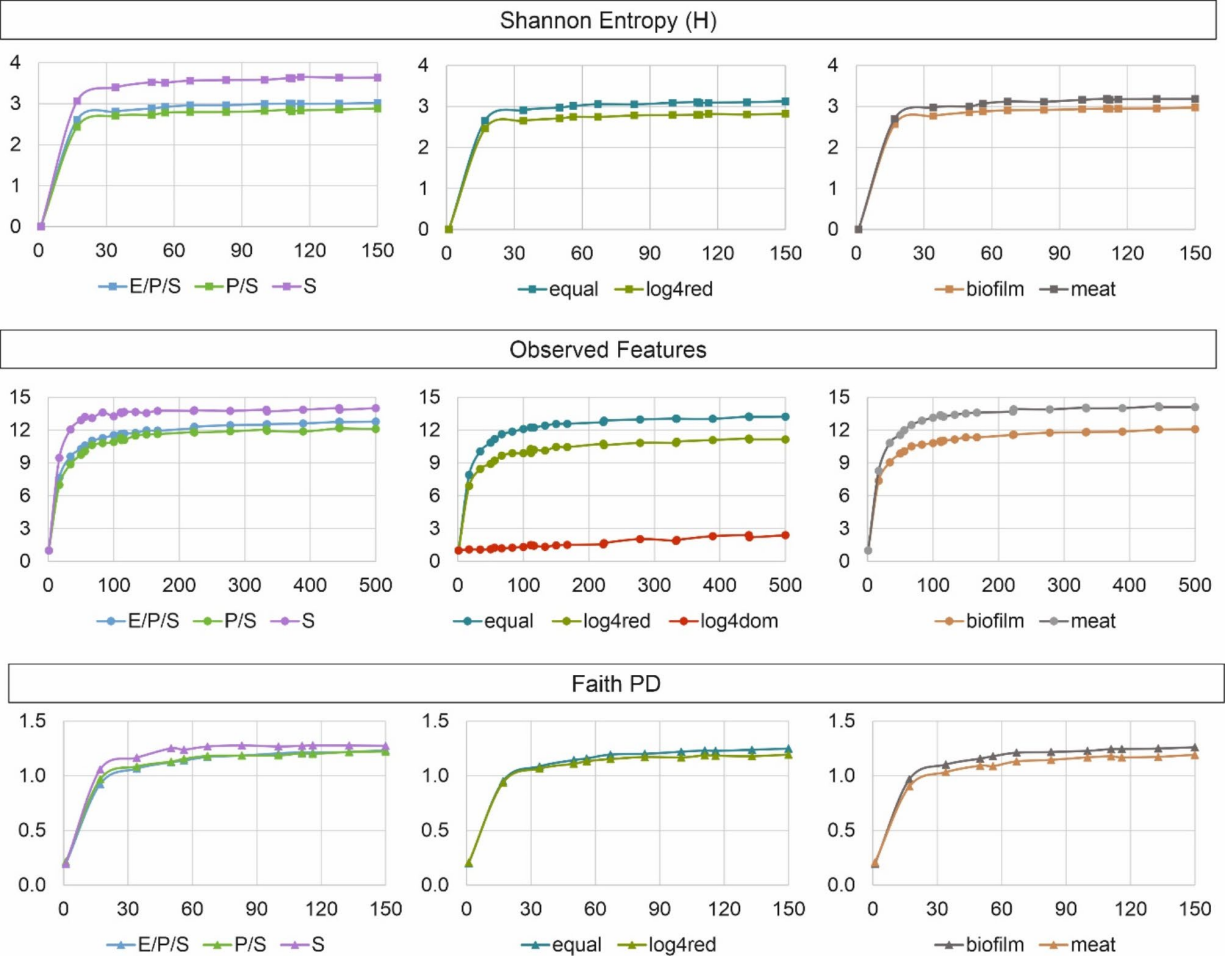
Rarefaction curves for the alpha diversity measures Shannon Entropy, Observed Features, and Faith PD displaying the influence of different factors. These are DNA extraction (E), PCR amplification (P), sequencing (S) (combined as E/P/S and P/S, or S alone), uniform species distribution (equal) vs. dominance (log4dom) or dilution (log4red) of single species, as well as differences between biofilm and mock community samples. OTU clustering was conducted at 98.7% identity for biofilm samples, while a cutoff of 99.5% was used for meat samples

Selected Rarefaction curves for Shannon Entropy, Observed Features and Faith PD are depicted in Fig. 3. All curves confirm that maximum values were reached at a sequencing depth of 15 to 150 reads for the equal and log4red samples. While there was no difference in curve progression between the factors extraction/PCR/sequencing (E/P/S), PCR/sequencing (P/S) and sequencing (S), overall higher values were reached by the samples testing for the impact of sequencing alone. Maximum Observed Features (i.e. OTUs) values were higher for the samples containing uniform isolate concentrations, and higher for the meat mock community containing 14 isolates compared to biofilm mock communities containing 13 isolates. For the samples containing *A. guillouiae* M9 in dominance of 4 log_10_ cfu/ml (log4dom), an Observed Features value of six was reached only after a sequencing depth of 4500 (data not shown).

**Fig. 4:**
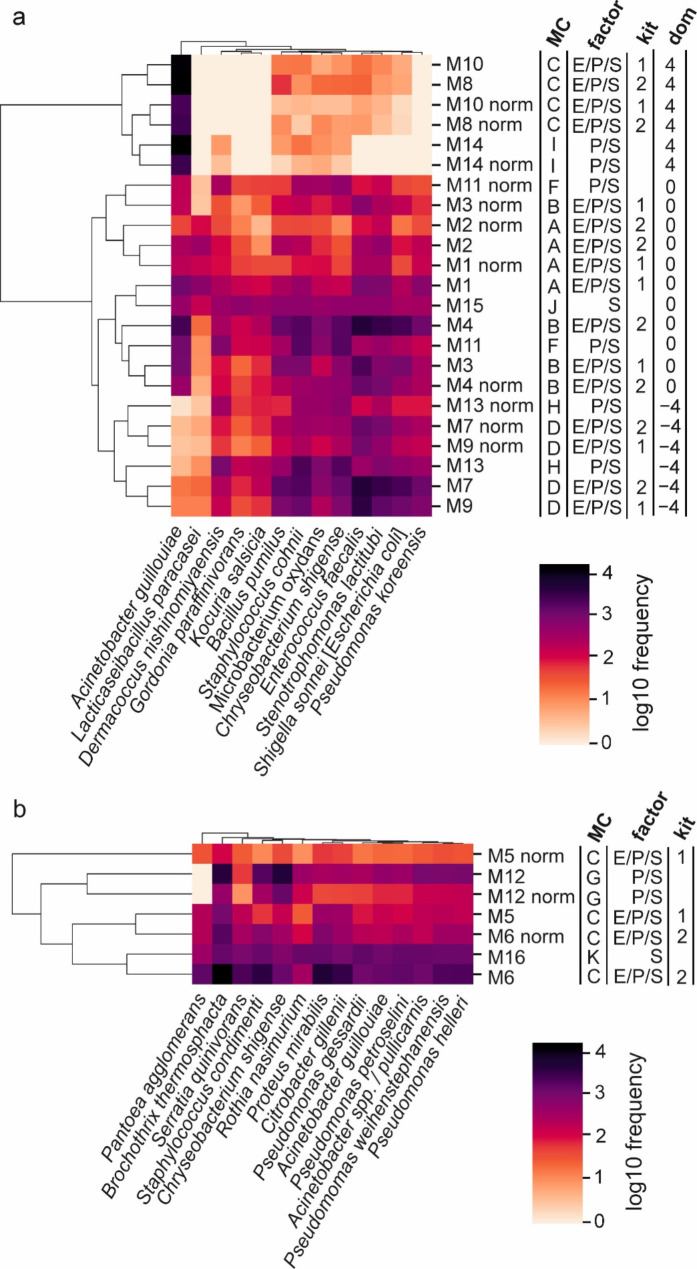
Heatmaps depicting absolute species abundances for biofilm (**a**) and meat (**b**) mock communities. Relevant factors and properties are indicated in the columns next to the sample names. MC, mock community; E, DNA extraction; P, PCR amplification of 16S rRNA genes; S, sequencing; dom, dominance of isolate *Acinetobacter guillouiae* M9 by the factor of 4/0/−4 log_10_

Absolute read frequency per species is displayed by heatmaps in Fig. 4. Samples with similar composition are expected to cluster together, given their overall read frequencies are comparable to each other. This is clearly evident for the samples testing for dominance (log4dom) or dilution (log4red) of the *Acinetobacter guillouiae* M9 isolate by the factor of 4 log_10_ or − 4 log_10_, respectively, in biofilm mock communities (Fig. 4A). The dominance of the *A. guillouiae* M9 isolate is clearly reflected by the color pattern of the respective six samples, which form a separate cluster. Although *A. guillouiae* dominated these samples by the factor of 4 log_10_, seven (M8 + M10) and five (M14) other species were additionally detected in low frequencies, respectively. Four of those species belonged to the phyla *Actinomycetota* and *Bacillota*. *A. guillouiae* was also detected if diluted compared to other strains by the factor 10^− 4^ (M7, M9, M13). Normalized samples M2 (biofilm, Fig. 4A) and M5 (meat, Fig. 4B) appear in lighter colors due to their overall low read frequency. Uniform read distribution across all species is represented by almost absence of color gradation of the samples M15 (Fig. 4A) and M16 (Fig. 4B) testing for the impact of sequencing. In the case of M5 (Fig. 4B), M11 and M13 (Fig. 4A) 16S copy number normalization lead to clustering of the respective samples in distance to their parent samples.

**Fig. 5:**
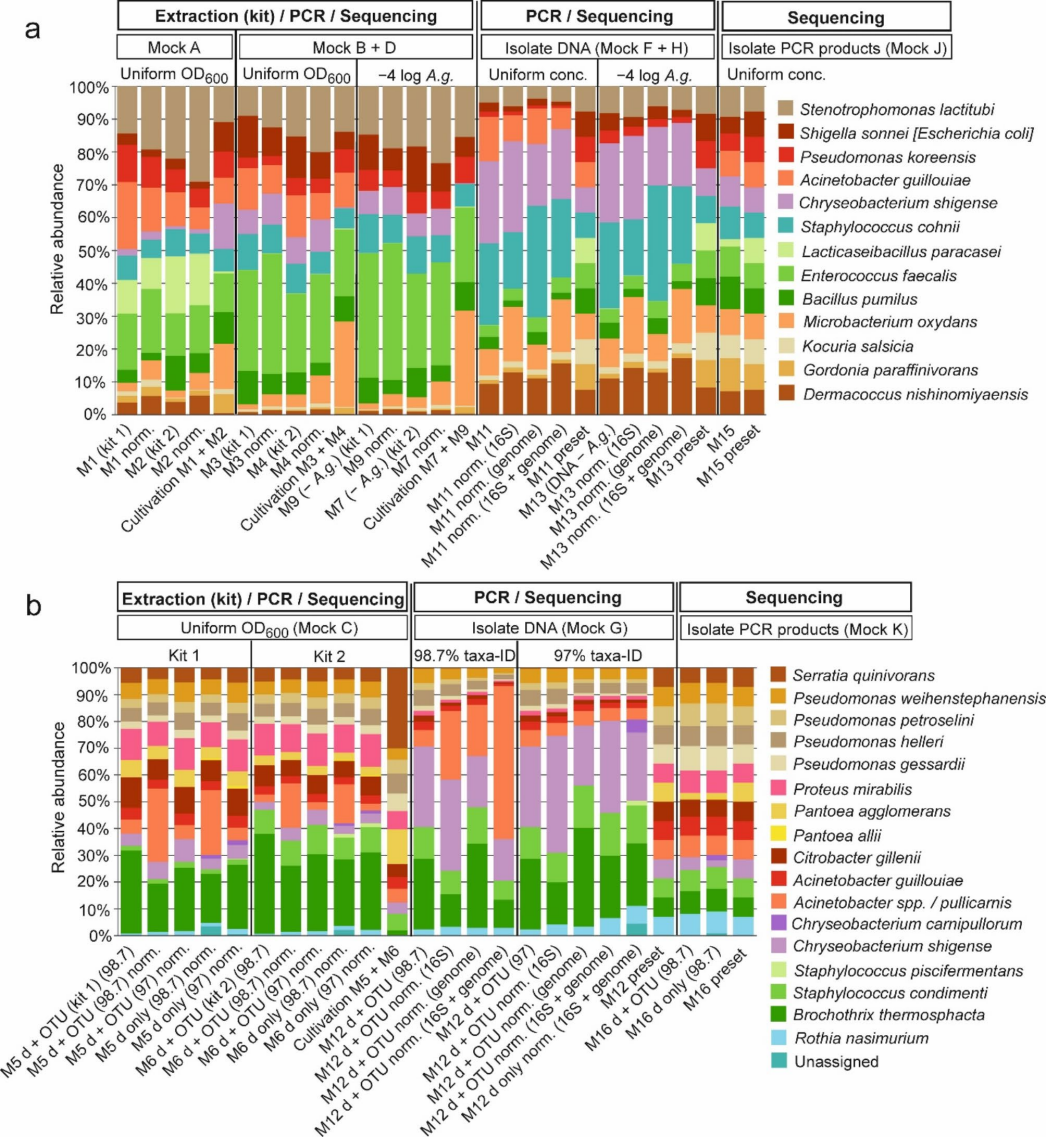
Taxa barplots comparing observed vs. preset species abundances. **a**, biofilm mock community; **b**, meat mock community. Norm, normalized for 16S rRNA gene copy number; − *A. g.*, dilution of isolate *Acinetobacter guillouiae* M9 by the factor of 10^− 4^; 16S, genome, normalization for 16S rRNA gene copies and genome size; d + OTU, denoised and OTU-clustered; d only, denoised; 97 vs. 98.7, percent sequence identity to reference sequences used for taxonomic classification

**Fig. 6:**
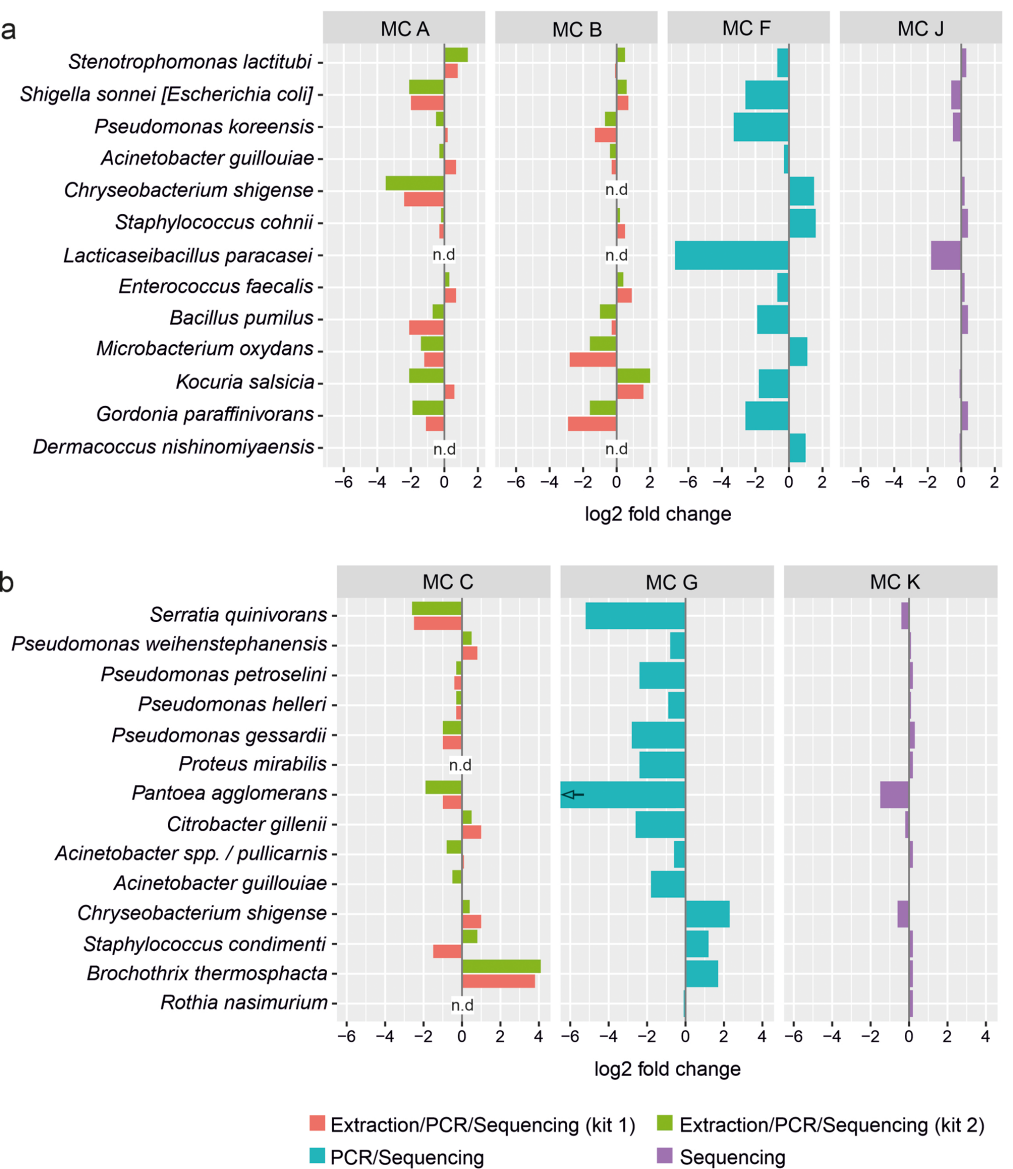
log2 fold-changes of observed compared to preset abundances. a, biofilm mock community (MC) samples; b, meat mock community (MC) samples. n.d, not determined because cultivation counts were below limit of detection; the arrow indicates that the fold change is lower than depicted, because no sequence reads of the isolate *Pantoea agglomerans* H6a were detected

Observed and preset community compositions are compared in the taxa barplots shown in Fig. 5A (biofilm) and 5B (meat). The isolates constituting the respective mock community are grouped by phylum: *Actinomycetota*, *Bacillota*, *Bacteroidota*, and *Pseudomonadota* (from bottom to top) to allow for detection of effects related to higher taxonomic ranks. Figure 6 visualizes the influence of different factors on the abundance of each species as log2 fold changes compared to the preset abundances. Overall, the phylum *Actinomycetota* was underrepresented in the samples after DNA extraction. Especially the species *Microbacterium oxydans* and *Gordonia paraffinivorans* were affected (Figs. 5A and 6A). *Lcb. paracasei* was detected in mock community A by sequencing, but not by cultivation. The same was true for *Chryeobacterium shigense* in mock community B (Fig. 5). For the meat mock communities the species *Brochothrix thermosphacta* was clearly overestimated by DNA extraction and PCR amplification procedures (Figs. 5B and 6B). PCR amplification of 16S rRNA genes produced the biggest deviation between preset vs. observed abundances (Fig. 6). Since uniform DNA concentrations of isolates were used as starting material, normalization for genome size was applied in addition to normalization for 16S rRNA gene copy numbers. In most cases, normalization for 16S rRNA gene copy number alone produced similar effects as additional normalization for genome size. One exception was the species *Staphylococcus cohnii*, where 16S copy number normalization led to decreased abundance, while genome size normalization produced increased abundance. Accordingly, combining both normalization procedures nearly restored the abundance detected in the unprocessed sample (Fig. 5A). These effects are caused by the high number of six 16S rRNA gene copies combined with a small genome size of 2.7 Mb of this species (Table 1). Overall, the species *C. shigense* was overrepresented in both mock communities after PCR amplification, despite different strains were used. In both mock communities, the genus *Staphylococcus* was also overrepresented after PCR amplification. The opposite was true for many species of the phylum *Pseudomonadota*. Especially the species *Escherichia coli*, *Pseudomonas koreensis* (Fig. 6A), *Serratia quinivorans*, and *Pantoea agglomerans* (Fig. 6B) were underrepresented after PCR amplification. For both biofilm and meat mock communities, sequencing produced the lowest overall bias. Most of the observed abundances matched with the preset abundances. However, *Lcb. paracasei* was clearly underrepresented in sample M15 (Figs. 5A and 6A). The same was true for *Pantoea agglomerans* in sample M16 (Figs. 5B and 6B).

To test the effect of the sequence identity limit for species assignment, we added isolate *Acinetobacter* spp. R25a to the meat mock community. The strain shows  ≤  98.2% 16S rRNA gene sequence identity to all deposited type strain sequences (Table 1), and was used to test the response of the bioinformatics pipeline to unknown taxa. When using 98.7% identity as the reference for species assignment, the sequence remained “unassigned”. The unassigned ASV/OTU remained unnormalized for 16S rRNA gene copy number, which led to overrepresentation in all normalized samples using 98.7% taxa-ID for species delineation (Fig. 5B). When using 97% identity the sequence was assigned to *Acinetobacter pullicarnis* and subsequent copy number normalization was conducted. For the meat mock community the effect of denoising without subsequent OTU-clustering reported the presence of three additional species which were not part of the mock community: *Pantoea allii*, *Chryseobacterium carnipullorum*, and *Staphylococcus piscifermentans*, together with the appearance of unassigned ASVs, all with low abundances (Fig. 5B).

## Discussion

Rarefaction analysis confirmed that the diversity of mock community samples was covered within all samples despite of varying read frequencies. While 15–150 reads were necessary to reach maximum alpha diversity measures (Fig. 3), read frequency exceeded 509 (M2 norm.) in all samples (Table 2). However, mock communities contained only 13 (biofilm) and 14 (meat) species, respectively, which is expected to be a much lower diversity compared to real food samples [25, 40]. Consequently, sequencing depth should be chosen depending on the expected diversity within the foodstuff of interest. If rare sequences are to be detected, PacBio recommends to apply higher sequencing depths of > 6,000 [41]. We demonstrated that rarefaction analysis is an appropriate tool for determining required read numbers. This becomes clear from the samples with dominance of *A. guillouiae* M9, were rarefaction analysis indicated saturation only after 4500 reads. Sequencing depth is an important parameter in food-associated habitats, especially with low bacterial loads, since host DNA of animals and plants may be co-extracted in large amounts. Although 16S rRNA amplicon sequencing using bacteria-specific primers will reduce this problem to some extent, mitochondria and chloroplast reads might still be present in the dataset [11]. Eukaryotic reads can be sorted out bioinformatically, but will reduce sequencing depth for target prokaryotic sequences if still present in the library. Thus, it should be best practice to exclude host DNA already prior to PCR.

Normalization of data on the basis of 16S rRNA gene copies per cell considers the large differences in 16S rRNA gene copy numbers for different bacterial taxa, and is easy to conduct by using the gcn-norm copy-num-normalize plugin [33]. For the biofilm mock community, minor effects on overall community composition were observed, which is in line with the findings of other authors [9]. However, several issues might occur with copy number normalization, which is exemplified by the meat mock community (Fig. 5B). If ASVs/OTUs are unassignable to reference sequences contained in the database, they will be normalized by the factor of one, while all other ASVs/OTUs will be normalized by higher numbers according to their species or genus taken from the rrnDB [32]. Consequently, unassigned ASVs/OTUs will be massively overrepresented in the abundance table after copy number normalization. This was demonstrated with the strain *Acinetobacter* spp. R25a in the meat mock community when using 98.7% taxa-ID for species-assignment, as the cutoff for species identity [35]. The presence of sequences from novel species displaying less than 98.7% identity to known type strains is a realistic scenario when analyzing food samples. One solution is choosing lower % taxa-IDs for species assignment, as it was done with 97% in the meat mock community samples (Fig. 5B). In this case, it is advisable to inspect taxonomic assignment manually to ensure detection of potentially novel species. Another possibility is to choose a maxaccepts value higher than one in the “feature-classifier classify-consensus-blast” command, e.g. five accepted hits as recommended by the program. However, if not all hits belong to the same species, the sequence will be assigned to the next common higher taxonomic rank (i.e. genus or family) [37]. This is appropriate for short-read amplicon sequencing data investigating hypervariable regions of around 450 bp length [19]. However, a strength of long-read sequencing analysis is that the complete V1-V9 region of the 16S rRNA gene (1,500 bp) is covered, allowing for taxonomic assignment at species level. Consequently, parameters chosen for Illumina-based analysis are not always best practice for long-read sequencing approaches. D’Amore et al. [22] and Notario et al. [24] found that PacBio data match well with short-read data when sequencing the same mock community samples. Species assignment is more reliable compared to short-read data. Notario et al. [22] even found variations in taxonomic assignments when sequencing different hypervariable regions while using the same short-read platform. To assess the possibility to detect even taxonomically close groups, four different species of the genus *Pseudomonas* were added to the meat mock community. All four species were assigned to unique ASVs/OTUs (Figs. 4B and 5B), and were correctly identified. This was only possible using the full-length 16S rRNA gene sequence. This is of particular importance since spoilage potential as well as pathogenic potential is species specific for the genus *Pseudomonas*, which is also true for many other genera.

Each ASV/OTU was assigned to the expected species within the mock community with ≥ 99.8% sequence identity compared to the respective isolate 16S rRNA sequence generated by Sanger sequencing (data not shown), which underlines the high quality of PacBio ccs-reads. Correct taxonomic assignment is strongly influenced by the choice of database. Commonly applied 16S rRNA gene reference databases are SILVA [42] and Greengenes2 [43]. However, not all SILVA records contain species-level taxonomy, and species-level taxonomy is not curated [24], making this database less suitable for full-length 16S rRNA gene sequencing approaches. Using a custom RefSeq database [36] renders the amplicon sequencing approach comparable to identification of Sanger-sequenced bacterial isolates using the NCBI BLAST website [34]. The suitability of our approach was confirmed by detection of all members of the mock communities, while other authors found that no database was able to detect all members of mock communities when performing short-read amplicon sequencing [20, 24]. It should be noted, however, that choice of database always depends on the aim of the study. Moreover, different versions of a database may produce varying results. Thus, it is advisable to use up-to-date databases containing also sequences of only recently described species.

There is no consensus in the literature about directly using ASVs, e.g. those resulting from the denoising process of DADA2, for taxonomic assignment, or performing additional OTU clustering to reduce the amount of features [3, 43, 44]. For this reason, we compared the microbial compositions of meat mock communities using both approaches (Fig. 5B). If only denoised data were used for taxonomic assignment without subsequent OTU-clustering, species diversity was overestimated due to the appearance of additional species of the genera *Chryseobacterium*, *Pantoea*, and *Staphylococcus*. Additionally, unassigned ASVs appeared which have the potential to cause bias if copy number normalization is performed (see above). Besides OTU-clustering, amount of ASVs was reduced by filtering for frequency and appearance in more than one sample. It is advisable to perform filtering prior to taxonomic assignment to sort out artifacts and contaminants [40]. However, stringency of filtering parameters strongly depends on the objective of the study. If the microbial load of the sample is low, or if rare species are to be detected, frequency filtering can be omitted.

The heatmaps depicted in Fig. 4 show that dominance of the *Acinetobacter guillouiae* M9 isolate by the factor 10,000 in the biofilm mock community reduced the number of detected species to eight out of 13. Dilution of *A. guillouiae* by the same factor did not fully inhibit the detection of this species, although its abundances were below 0.1%. Determining cultivation counts of mock communities, uniform colony morphology was observed for the dominance samples, while *A. guillouiae* colonies were not detected after dilution (data not shown). This suggests that 16S rRNA gene amplicon sequencing was more sensitive in detecting minor species present within a habitat compared to cultivation. Since species in foodstuff are not expected to be equally distributed, it is valuable to know that also minor species can be detected, in spite the presence of dominating taxa.

We had two assumptions when conducting the study. First, we assumed that Gram-positive cells, i.e. the phyla *Actinomycetota* and *Bacillota*, are harder to disrupt [44], resulting in lower DNA concentrations and thus lower abundance in samples testing for the influence of DNA extraction and subsequent PCR-amplification (M1-M10). For this reason, two different DNA extraction kits were tested for differences in their efficiency to extract the DNA from all isolates contained in the mock communities. The second hypothesis was that mol% G + C content of the 16S rRNA genes would affect efficiency of PCR amplification. We hypothesized that low percentages would result in preferred amplification due to lower melting temperatures, and vice versa. The phylum *Actinomycetota* has a high DNA mol% G + C content [45], which was also the case for the 16S rRNA genes of isolates added to biofilm and meat mock communities (Table 1). Irrespective of the mock community of interest, log2 fold-changes of PCR/sequencing alone and DNA extraction/PCR amplification/sequencing pointed in the same direction for most taxa (Fig. 6). Since biofilm communities A and B (MC A and MC B) can be regarded as biological duplicates, inter sample variability can be concluded from Fig. 6A. No significant difference was observed between DNA extraction kits. As a conclusion, DNA extraction itself seemed to exert minor effects on taxa relative abundance, which leads to the conclusion that cell disruption was successful for all isolates. The impact of DNA extraction varied in different studies. While some studies also came to the conclusion that DNA extraction had only minor effects on the observed microbial community composition [18], other studies found differences due to DNA extraction [8, 10, 46]. The choice of DNA extraction kit is strongly dependent on the sample of interest. DNA extraction from complex food matrices might be more challenging than from liquid growth medium used in the present study. Foodstuff contains various amounts of lipids, polysaccharides, and proteins, all of which may hamper efficiency of extraction. Thus, it is recommended to evaluate the extraction process using bacterial taxa representative of the food matrix [4]. Optimal extraction procedures may additionally involve mechanic lysis such as bead beating instead of or in addition to enzymatic approaches. The effect of DNA extraction and PCR amplification seemed to be species dependent for isolates of the phylum *Actinomycetota*, although a tendency was observed that most species were underrepresented. Underrepresentation was expected due to decreased lysis efficiency combined with high mol% G + C contents of their 16S rRNA genes. However, the genera *Microbacterium* and *Dermacoccus* were even overrepresented in samples testing for the impact of PCR amplification/sequencing alone (Figs. 5A and 6A). Species of the phylum *Bacillota* were expected to be overrepresented in samples testing for the effect of PCR amplification/sequencing alone owing to their low mol% G + C content of their 16S rRNA genes (Table 1). This effect was observed for the genera *Brochothrix* and *Staphylococcus* in meat mock community samples, and additionally for the genus *Chryseobacterium* belonging to the phylum *Bacteroidota* (Figs. 5B and 6B). When plotting mol% G + C content of 16S rRNA genes against deviation from expected abundance for each species, we detected a significantly negative correlation (R² = 0.415, *p* = 0.013) for the meat mock community, while the correlation was not as pronounced and insignificant for the biofilm mock community (R² = 0.175, *p* = 0.154). As a conclusion, preferential amplification of sequences is dependent on the overall variation of mol% G + C contents of 16S rRNA genes within a community. If the variation is high, the overall effect on single taxa might be lower compared to communities with the presence of one or few species with remarkably lower mol% G + C contents (i.e. *C. shigense* H9a in the meat mock community). In many cases, negative log2 FCs were observed for species belonging to the phylum *Pseudomonadota*, which was unexpected since cells should be easy to lyse and did not display elevated mol% G + C contents compared to the mean values (Table 1). It was observed that rigorous lysis might lead to underrepresentation of Gram-negative taxa, which was attributed to the loss of DNA due to early cell disruption combined with DNA shearing [2, 27]. However, for most species in the current study PCR amplification seemed to be responsible for the observed effect, since samples testing for the effect of PCR amplification/sequencing alone showed drastic effects on the abundances (Fig. 6). The primary goal of the current study was to compare preset vs. observed abundances of the same sample to assess the sources of bias researchers should be aware of when interpreting long-read PacBio amplicon sequencing data. Comparing the biological duplicates M1/M3 and M2/M4, no major inter-sample variabilities were observed that outperform the effects caused by the parameters analyzed in this study. Future studies should confirm our findings by incorporating biological replicates as part of sequencing sample batches. As shown here, this point is especially relevant when testing the complete workflow starting from DNA extraction.

Overall, the effect of DNA extraction alone was hard to assess for certain strains, as difficulties in cultivation resulted in problems assessing the expected community composition. At the same time, the detection of isolate sequences for which cultivation was unsuccessful proved 16 S rRNA gene amplicon sequencing as a suitable tool for detecting species that are difficult to cultivate. Xue & Marco [27] compared cultivation and microscopic cell counts to amplicon sequencing data and found microscopy to be more representative of the expected value, making it a suitable alternative to colony counting. Owing to the low comparability between cultivation and molecular methods, we recommend using a combination of methods to determine the overall community composition of a habitat.

Concluding from our observations, PCR amplification of 16S rRNA genes seemed to produce the strongest bias on the observed community composition while sequencing alone was nearly able to reproduce the expected abundances of both mock communities. As a consequence, quantitative community composition assessed by 16S rRNA gene amplicon sequencing should be interpreted with caution, since it will not reflect the actual abundance of taxa within the habitat of interest. Other authors also observed insufficient comparability of species abundances determined by 16S amplicon sequencing compared to the actual species composition of mock communities [16, 18, 20, 22]. It is advisable to analyze easy to handle mock communities containing few representative species of the habitat of interest to evaluate the effect of DNA extraction, PCR amplification of 16S rRNA genes and sequencing on certain taxa. Inter-sample variability should be addressed by analyzing biological replicates. Metagenome sequencing is an alternative to assess microbial community composition without amplification of 16S rRNA genes. In addition to less biased microbial composition, metagenome sequencing will not only detect bacteria, but also viruses, and fungi, leading to a more throughout assessment of the habitat’s microbiome. Moreover, metabolic functions can be inferred from the genes detected by metagenome sequencing [21, 23]. Metagenome sequencing is more expensive and requires large amounts of high-quality DNA, which may limit its application, e.g. in habitats with low microbial loads [3]. If metagenome sequencing is not applicable, template concentration as well as PCR cycles can be adjusted to the necessary minimum to minimize the effect of preferred amplification as well as chimera formation [22, 47]. However, owing to the exponential nature of PCR amplification, less abundant taxa could be discriminated and would remain undetected, despite substantially influencing the ecology of the respective habitat.

## Conclusions

In the present study we used two mock communities representing milking machine biofilms and meat to assess the impact of DNA extraction, PCR amplification of full-length 16S rRNA genes, sequencing, dominance and dilution of single species, as well as bioinformatics analysis on the observed community composition and alpha diversity using PacBio long-read sequencing. We demonstrated that PCR amplification of 16S rRNA genes produced the strongest bias on the observed outcome, which was most likely attributed to diverging amplification efficiencies due to varying mol% G + C contents of the 16S rRNA genes. The observed results demonstrate that relative abundances taken from 16S amplicon data are not representative of the habitat’s actual microbiome. Thus, it is recommended to evaluate 16S amplicon results carefully, e.g. by parallel metagenome analysis or cultivation. Moreover, bioinformatics workflows including databases and thresholds for species assignment should be benchmarked using mock communities representative of the habitat of interest, and subsequently stay consistent during the analysis.

## Electronic supplementary material

Below is the link to the electronic supplementary material.

Supplementary Material 1

## Data Availability

Nearly full-length 16 S rRNA gene sequences of mock community isolates generated by sanger sequencing are deposited in GenBank under the accessions PP905163 to PP905189. Raw amplicon sequencing data is deposited in the sequence read archive (SRA) as BioProject PRJNA1122981 with BioSample Accessions SAMN41796194 to SAMN41796209. The custom Refseq 16 S rRNA gene database used for species assignment in this study, containing 24,925 type strain sequences with lengths ≥ 1200 bp can be accessed via https://github.com/baer-mareike/Refseq-16S-database.
