# Supplementary material for: Impact of DNA extraction, PCR amplification, sequencing, and bioinformatic analysis on food-associated mock communities using PacBio long-read amplicon sequencing
Source: BMC Microbiol. 2024 Dec 6;24:521. doi: 10.1186/s12866-024-03677-8 (PMC11622462; doi:10.1186/s12866-024-03677-8)
Supplement: Supplementary file 1 — Supplementary Material 1 [file 12866_2024_3677_MOESM1_ESM.docx]

**Impact of DNA extraction, PCR amplification, sequencing, and bioinformatic analysis on food-associated mock communities using PacBio long-read amplicon sequencing**

Mareike Baer^a 1)^, Lisa Höppe^a^, Waldemar Seel^b^, André Lipski^a^

^a^ University of Bonn, Institute of Nutritional and Food Sciences, Food Microbiology and Hygiene, Friedrich-Hirzebruch-Allee 7, 53115 Bonn, Germany

^b^ University of Bonn, Institute of Nutritional and Food Sciences, Nutrition and Microbiota, Katzenburgweg 7, 53115 Bonn, Germany

^1)^ Corresponding author: mabaer@uni-bonn.de

**Supplementary Material**

| **Sample-ID** | **Input reads** | **Filtered, denoised, non-chimeric** | **% of input** | **Freq. after norm.** | **% of input** |
| --- | --- | --- | --- | --- | --- |
| M1 | 18207 | 13972 | 76,7 | 3055 | 16,8 |
| M2 | 7006 | 5281 | 75,4 | 1166 | 16,6 |
| M3 | 25768 | 19415 | 75,3 | 4065 | 15,8 |
| M4 | 75506 | 55397 | 73,4 | 12346 | 16,4 |
| M5 | 4700 | 3481 | 74,1 | 509 | 10,8 |
| M6 | 61849 | 44686 | 72,3 | 6452 | 10,4 |
| M7 | 63625 | 46564 | 73,2 | 10680 | 16,8 |
| M8 | 77627 | 53685 | 69,2 | 7686 | 9,9 |
| M9 | 36532 | 27648 | 75,7 | 6305 | 17,3 |
| M10 | 60141 | 45294 | 75,3 | 6477 | 10,8 |
| M11 | 36439 | 19100 | 52,4 | 4646 | 12,8 |
| M12 | 36761 | 17146 | 46,6 | 3125 | 8,5 |
| M13 | 42631 | 21918 | 51,4 | 5612 | 13,2 |
| M14 | 77800 | 55135 | 70,9 | 7880 | 10,1 |
| M15 | 19150 | 13400 | 70,0 | n.a. | |
| M16 | 24503 | 17316 | 70,7 | n.a. | |
